# Pectoralis major myocutaneous versus pectoralis major myofascial flap for type III and type IV tongue defects reconstruction in patients operated for squamous cell carcinoma: A protocol

**DOI:** 10.6026/973206300223480

**Published:** 2026-06-30

**Authors:** Karamvir Karamvir, Nitin Bhola

**Affiliations:** 1 Department of Oral & Maxillofacial Surgery, Sharad Pawar Dental College & Hospital, Wardha, Maharashtra, India

**Keywords:** Pectoralis major myofascial flap (PMMC), pectoralis major myocutaneous flap (PMMF), tongue squamous cell carcinoma (TSCC)

## Abstract

Reconstruction following extensive resection of tongue squamous cell carcinoma remains challenging because the optimal pedicled flap for achieving superior functional recovery and aesthetic outcomes is still uncertain. Therefore, it is of interest to compare pectoralis major myocutaneous (PMMC) and pectoralis major myofascial (PMMF) flaps for reconstruction of Type III and Type IV tongue defects in patients undergoing surgical treatment for squamous cell carcinoma. Twenty-four eligible patients will be randomly allocated into two groups, and post-operative outcomes including flap-related complications, tongue shape, swallowing, speech and tongue mobility will be evaluated at 1 week, 1 month and 3 months using standardized clinical assessment scales. Functional and aesthetic outcomes between the two reconstruction techniques will be analyzed using appropriate statistical methods to determine differences in clinical performance and patient rehabilitation. Thus, data shows to generate evidence that supports selection of the most effective pedicled flap for tongue reconstruction and improves functional recovery, quality of life and clinical decision-making in resource-constrained settings.

## Background:

Oral cancer remains a major health burden in India, ranking among the most common malignancies and being reported in nearly 25% of cancer registries. Alarmingly, about 80% of cases are diagnosed at advanced stages, limiting survival [1]. GLOBOCAN data indicate that India contributes to one-third of global oral cancer cases, with oral cancer accounting for approximately 30% of all cancers nationally. ICMR reports show that men are disproportionately affected, largely due to tobacco and alcohol use [2]. Tongue squamous cell carcinoma (TSCC) constitutes nearly 45% of oral cavity cancers [3], with an increasing prevalence among younger individuals [4, 5, 6, 7]. Its aggressive biology, early metastasis and poor prognosis emphasize the need for timely diagnosis, effective therapies and reconstruction to improve survival and quality of life. Enhancing therapeutic approaches tailored to younger patients may help improve their prognosis and overall survival rates. With the increasing burden of TSCC, particularly among young individuals, prioritizing research and clinical advancements in this area is essential for better management and outcomes [8]. The tongue, an essential organ within the oral cavity and oropharynx, is a complex muscular structure covered by a thick mucous membrane. Although primarily responsible for taste perception, the tongue also plays a crucial role in mastication, deglutition, articulation and oral hygiene. It is innervated by five cranial nerves, which contribute to its diverse functions. The tongue is divided into the tip, body and base, with complex innervation supporting its functions [9]. Tobacco use remains the most significant risk factor for developing squamous cell carcinoma of the tongue, with smoking and smokeless tobacco products contributing to approximately 85% of head and neck cancers. Secondhand smoke exposure also increases the risk. Additionally, alcohol consumption is a notable factor, particularly when combined with tobacco, leading to a synergistic effect that exponentially increases the likelihood of cancer development [10]. Other contributing factors include poor oral hygiene, tooth loss and, potentially, the prolonged use of alcohol-based mouthwashes. Dietary habits influence oral cancer risk, with vitamin A, β-carotene and α-tocopherol offering protective effects [11]. Genetic predisposition also contributes, as inherited syndromes such as Fanconi anemia and dyskeratosis congenital impair DNA repair and increase the risk of malignancy, including oral cancers [12, 13, 14]. HPV's role in tongue cancer is less clearly defined than in oropharyngeal cancers, but established carcinogens, including tobacco, alcohol and betel nut, remains major factors, emphasizing the importance of preventive strategies [15]. Over 90% of oral cancers do squamous cell carcinomas, highlighting the importance of risk assessment, understand pathogenesis and early detection for better outcomes. Surgical excision is the primary treatment but often causes significant tissue loss. Reconstruction using pedicled pectoralis major myocutaneous (PMMC) flaps or microvascular free tissue transfer is essential to restore function and aesthetics. The PMMC flap gained popularity because of its technical simplicity, reliability and close proximity to the head and neck region. Despite the earlier introduction of microvascular free flaps, they were not widely adopted because of the need for specialized training and infrastructure [16]. The selection of a reconstruction method depends on the defect size and follows the reconstructive ladder. In advanced OSCC cases, reconstructive options primarily include pedicled flaps and microvascular free flaps (MFFs). MFFs have revolutionized reconstruction by enabling the repair of complex oral cavity defects, significantly enhancing both function and appearance and thereby improving patient outcomes. However, MFFs carry the risk of flap failure due to factors such as inadequate surgical expertise, flaws in flap design and harvesting, vascular anastomosis issues and complications at irradiated donor sites.

Venous congestion (83%) and arterial compromise (8%) are common complications. Additionally, MFFs may not always be feasible because of financial constraints or limited healthcare resources [17]. Pedicled flaps, such as the pectoralis major myocutaneous (PMMC) flap, remain a reliable option for oral cavity reconstruction because of their ease of harvest, accessibility and relatively low failure rates. They are particularly valuable in high-volume centers with long surgical waiting lists. However, in female patients with advanced OSCC, bulky breast tissue and breast contour deformities may limit their application, making the pectoralis major myofascial (PMMF) flap a practical alternative for large oral cavity defects [18]. One major drawback of the PMMC flap is its bulk, which is influenced by the subcutaneous fat overlying the muscle and can hinder speech and swallowing rehabilitation, especially in anterior oral cavity and pharyngeal sites. This issue is more problematic in female or broad-chested patients and complications may be worsened when the mandible is preserved. Other limitations include intraoral hair growth, chest wall deformities and technical challenges with pedicle rotation. The PMMF flap, consisting of the pectoralis muscle and fascia without a skin island, addresses many of these drawbacks and has been reported as a versatile reconstructive option, although it remains underrepresented in the literature [19]. Partial hypopharyngeal defects following total laryngectomy (TLE) also pose reconstructive challenges. Loss of speech, impaired swallowing and complications such as fistulas, especially in previously irradiated patients, demand tailored approaches to optimize outcomes. While many techniques use free or regional flaps to create a cutaneous lining, the PMMC flap has lost favor because of its bulk and poor pliability. To overcome these issues, Robertson and Robinson introduced the PMMF flap in the 1980s, reducing complications. However, comparative evidence between PMMC and PMMF flaps remains limited [20]. Our study evaluates the post-operative outcomes of both flaps in tongue reconstruction. Most reconstructive techniques involve inverting a myocutaneous or fasciocutaneous free flap onto the remaining pharyngeal mucosa, forming a cutaneous lining. Historically, the pectoralis major myocutaneous (PMMC) flap has been widely used, but its bulkiness, poor pliability and risk of complications have reduced its popularity. To address these issues, Robertson and Robinson proposed the pectoralis major myofascial (PMMF) flap in the 1980s, which eliminates the skin island and reduces complications. Despite promising results, comparative studies between PMMC and PMMF flaps remain limited [20]. Therefore, this study aims to compare and evaluate the functional and aesthetic outcomes of the Pectoralis Major Myocutaneous (PMMC) flap versus the Pectoralis Major Myofascial (PMMF) flap for Type III and Type IV tongue defect reconstruction in patients operated on for squamous cell carcinoma of the tongue. Oral squamous cell carcinoma (OSCC) represents a major health burden in India, accounting for a significant proportion of global head and neck cancer cases. Advanced-stage disease often requires extensive surgical excision followed by reconstruction to restore function and aesthetics. In many developing and resource-limited settings, microvascular free flap reconstruction may not be feasible because of financial limitations, lack of infrastructure and patient-related factors. Under such circumstances, pectoralis major myofascial (PMMF) and pectoralis major myocutaneous (PMMC) flaps serve as reliable alternatives for reconstruction. Studies have shown that PMMF flaps are associated with lower complication rates and provide satisfactory functional and cosmetic outcomes, particularly in female patients, in whom preservation of breast contour is important [17]. PMMC flaps, based on the thoracoacromial artery, continue to play a significant role in head and neck reconstruction because of their proximity to the operative site, technical simplicity and ability to reconstruct complex defects effectively. They are especially useful in economically disadvantaged and malnourished patients, offering high success rates despite moderate complication rates [21]. Although traditional pectoralis myocutaneous flaps may present drawbacks such as chest wall deformity, hair growth and excessive tissue bulk, the development of pectoralis myofascial flaps has helped overcome many of these limitations. By eliminating the skin paddle, these flaps provide reduced bulk, improved cosmetic outcomes and reliable healing, with successful epithelialization occurring within a short period, even in patients undergoing radiotherapy [22]. Tongue squamous cell carcinoma (TSCC) has traditionally been associated with older individuals with long-term tobacco exposure. However, recent evidence indicates an increasing incidence among younger patients without conventional risk factors, suggesting that TSCC in younger populations may represent a distinct biological entity. Most studies define younger patients as individuals below 40 years of age, with a slight male predominance. While tobacco use remains a major risk factor in older adults, more than half of younger patients with TSCC have no history of smoking or tobacco use, indicating the possible involvement of alternative etiological factors such as genetic predisposition, viral infections and lifestyle-related influences. These findings highlight the importance of further research to identify nontraditional risk factors and improve prevention and early diagnosis strategies for younger populations affected by TSCC [8]. Oral cavity and pharyngeal cancers continue to contribute significantly to the global cancer burden, with higher incidence rates reported in men than in women. Management of oral tongue cancer requires a multidisciplinary approach involving oncologists, speech therapists, rehabilitation specialists and psychological support to address the complex functional and aesthetic challenges associated with treatment [15]. In reconstructive management following ablative surgery for oral squamous cell carcinoma, both Pectoralis Major Myocutaneous (PMMC) flaps and free flaps have demonstrated comparable complication rates. Although free flaps offer superior versatility and functional outcomes, PMMC flaps remain a practical and cost-effective option in resource-limited settings, particularly where financial constraints, lack of microvascular facilities and patient comorbidities limit the use of free flap reconstruction. Therefore, PMMC reconstruction continues to maintain clinical relevance in developing healthcare systems [16]. Therefore, it is of interest to compare the functional and aesthetic outcomes of pectoralis major myocutaneous and pectoralis major myofascial flaps for reconstruction of Type III and Type IV tongue defects following surgical treatment of tongue squamous cell carcinoma.

## Materials and Methods:

The study will be a parallel group randomized trial. It will be conducting from February 2025 to September 2026. This clinical study will take place at the Department of Oral and Maxillofacial Surgery, Siddharth Gupta Memorial Cancer Hospital, Sawing, Wardha. The research will be carried out under the auspices of the Datta Meghe Institute of Higher Education and Research, Sawing, Wardha Institutional Ethics Committee (IEC) approval from Datta Meghe Institute of Higher Education and Research (DMIHER) (DU) has been obtained and the process will begin after explaining the detailed treatment protocol to the patients. A total of 24 patients will be assessed for biopsy-proven cases of SCC, with 12 patients meeting the criteria for the study.

## Sample size calculation:

The sample size was calculated using Cochran's formula Considering a Z value of 1.96, a prevalence (P) of total flap necrosis reported as 2% (0.02) and a desired margin of error (e) of 8% (0.08), the formula was applied as:

n=z^2^(pq)/e^2^

n = 1.962 *0.02*(1-0.02)/0.08*0.08

= 11.76

Thus, a minimum of 12 patients were required for the study in each group (Total=12x2=24). The prevalence data were obtained from the study conducted by Milenovic *et al.* [22]. After diagnosis, patients will be divided into two groups by computer generated randomization and then alternatively select for the reconstruction with pectoralis major myocutaneous flap or pectoralis major myofascial flap.

## Inclusion criteria:

[1] All the patients age 18 years and above with Biopsy proven case of Squamous Cell Carcinoma of Tongue

[2] Type III and Type IV Tongue defects according to Ansari classification

[3] Patients fit to undergo surgery ASAI

## Exclusion criteria:

[1] All patients who are not willing to be a part of study

[2] All patients with uncontrolled systemic diseases

[3] Small lesions of tongue for primary closure.

[4] Poland Syndrome

## Study procedure:

Procedure will be done at Siddharth Gupta Memorial Cancer Hospital (SGMCH), DMIHER, Sawangi, Wardha for patients presenting with non- healing ulcers over the tongue.

[1] Patient Evaluation: Patients reporting to the Department of Oral and Maxillofacial Surgery will undergo a thorough examination.

[2] Case History: A detailed medical, dental and personal history will be documented.

[3] Clinical Examination: A comprehensive assessment of the oral cavity, focusing on the non-healing ulcer sites, will be conducted.

[4] Biopsy: A biopsy will be performed for histopathological examination to confirm the presence of squamous cell carcinoma.

[5] Radiographic Examination: Patients with confirmed squamous cell carcinoma will undergo a MRI to assess the extent of the lesion and detect any lymph node metastasis

This protocol ensures accurate diagnosis and appropriate management for patients presenting with suspected tongue cancer at SGMCH. After diagnosis, patients will be divided into two groups by computer generated randomization and undergo surgery under all aseptic protocols. After surgery, the patient will be monitored and after 1 week, 3 months and 6 months, they will be evaluated for functional and aesthetic outcomes, according to which the PMMC and PMMF flap will be judged. The two groups will be divided based on the type of reconstruction to be done after wide local excision or composite resection of the lesion and suitable neck dissection, which are 1) Group I - Reconstruction of surgical defect in squamous cell carcinoma of tongue with pectoralis major myocutaneous flap 2) Group II- Reconstruction of surgical defect in squamous cell carcinoma of tongue with pectoralis major myofascial flap. Once a pre-surgical workup has been completed, which includes pre-anaesthetic fitness, signed, showed and fully stated consents. After this pre-operative photos will be taken and the surgery will be performed. The conical assessment of the outcomes of the two types of reconstruction held would be done on the basis of the functional and aesthetic outcomes of the procedures post-operatively after 7 days, 1 month and 3 months of follow-up, which would help in keeping a close eye on the progress of the patients.

## Primary outcomes: 

Suture line leakage, Fistula, Flap discoloration and Donor site morbidity.

## Secondary outcomes: 

Aesthetic outcomes, including flap shape and size, along with functional outcomes such as swallowing ability, speech and tongue mobility, will be evaluated (Table 1, Table 2, Table 3, Table 4).

## Statistical analysis:

Following data collection, data will be entered into Microsoft excel worksheet (Microsoft, USA). Data analysis will done using IBM Statistical Package for Social Sciences (Statistics for Windows, Version 21.0. Armonk, NY: IBM Corp.) Categorical data will be described in terms of frequencies and percentages. Continuous data will be presented by mean and standard deviation (SD). Comparison of means to assess functional and aesthetic outcomes in reconstruction of Type III and Type IV Tongue defects with Pectoralis Major Myocutaneous Flap VS Pectoralis Major Myofascial Flap in patients being operated for squamous cell carcinoma of tongue will be done using Repeated Measures ANOVA and unpaired 't' test. For categorical data, chi square test will be used to compare the proportions. For analysis, p-value less than 0.05 will be considered statistically significant.

## Utility: 

The proposed study has significant clinical utility in guiding reconstructive decision-making for patients undergoing surgical management of tongue squamous cell carcinoma with Type III and Type IV tongue defects. By comparing pectoralis major myocutaneous flap and pectoralis major myofascial flap reconstruction, the trial will help identify the technique that provides better post-operative speech, swallowing, tongue mobility, tongue contour and overall functional rehabilitation.

## Expected outcome:

The present protocol is expected to determine whether PMMF flap reconstruction provides superior functional rehabilitation, improved speech and swallowing outcomes and reduced donor site morbidity compared to PMMC flap reconstruction in Type III and Type IV tongue defects.

## Conclusion:

We show that protocol compares PMMC and PMMF flaps for reconstruction of Type III and Type IV tongue defects after oncologic resection. The study is expected to identify the reconstruction technique that provides better functional recovery with fewer post-operative complications. The findings may strengthen evidence-based reconstructive planning and improve patient-centered outcomes in tongue squamous cell carcinoma surgery.

## Figures and Tables

**Table 1:** Tongue shape

**Tongue Shape**	**Score**
1. Well-shaped, reconstructed tongue	1
2. Near-normal shape but slightly altered	2
3. Broad triangular tongue	3
4. Small triangular tongue	4
5. Small, rounded remnant	5

**Table 2:** Swallowing scale

**Difficulty in Swallowing**	**Score**
1.Within normal limits	1
2.Minimal difficulty	2
3.Moderate difficulty	3
4.Severe dysphagia	4
5.Unable to swallow	5

**Table 3:** Speech scale

**Understand ability of speech**	**Score**
1. Always understandable	1
2. Near normal speech, occasional repetition	2
3. Necessary	
4. Usually understandable, face to face contact	3
5. Necessary	
6. Difficult to understand, use of actions necessary	4
7. Never understandable, might need to write for	5
8. Communication	

**Table 4:** Tongue mobility scale

**Tongue Mobility**	**Score**
1. Full, unrestricted tongue mobility in all directions. Normal swallowing and speech. No functional limitations.	1
2. Near-normal movement with slight restrictions (full protrusion and lateralization but slower, slight difficulty in elevation). Minimal impact on swallowing and speech.	2
3. Partial tongue mobility (protrusion beyond lower lip but incomplete, slow lateralization, elevation with effort). Swallowing and speech affected but functional with difficulty.	3
4. Limited tongue movement (<1 cm protrusion, poor lateralization, difficulty elevating). Significant swallowing and speech difficulties.	4
5. Tongue is immobile or has minimal movement. No effective protrusion, lateralization, or elevation. Severe difficulty in swallowing and speech.	5

## References

[1] Mohan P (2020). JCO Glob Oncol..

[2] Jeon JH (2017). Maxillofac Plast Reconstr Surg..

[3] Myers JN (2000). Otolaryngol Head Neck Surg..

[4] Khan Z (2025). Plast Reconstr Surg Glob Open..

[5] Siriwardena BS (2006). Oral Oncol..

[6] Costantino A (2023). Microsurgery..

[7] Mohideen K (2019). J Oral Maxillofac Pathol..

[8] https://www.ncbi.nlm.nih.gov/books/NBK507782/.

[9] Santos HB (2016). Med Oral Patol Oral Cir Bucal..

[10] Shklar G (1993). Nutr Cancer..

[11] Kutler DI (2003). Arch Otolaryngol Head Neck Surg..

[12] Alter BP (2009). Blood..

[13] Rosenberg PS (2005). Blood..

[14] Arrangoiz R (2018). Cancer Rep Rev..

[15] Padha K (2022). J Oral Biol Craniofac Res..

[16] Lech D (2024). J Clin Med..

[17] Dey S (2023). Indian J Otolaryngol Head Neck Surg..

[18] Tonsbeek AM (2023). J Plast Reconstr Aesthet Surg..

[19] Tonsbeek AM (2024). Cancers (Basel)..

[20] Sen S (2019). J Stomatol Oral Maxillofac Surg..

[21] Shindo ML (1992). Arch Otolaryngol Head Neck Surg..

[22] Milenovic A (2006). J Craniomaxillofac Surg..

